# Impact of Population and Pharmacogenetics Variations on Efavirenz Pharmacokinetics and Immunologic Outcomes During Anti-Tuberculosis Co-Therapy: A Parallel Prospective Cohort Study in Two Sub-Sahara African Populations

**DOI:** 10.3389/fphar.2020.00026

**Published:** 2020-02-07

**Authors:** Sabina Mugusi, Abiy Habtewold, Eliford Ngaimisi, Wondwossen Amogne, Getnet Yimer, Omary Minzi, Eyasu Makonnen, Christopher Sudfeld, Jürgen Burhenne, Eleni Aklillu

**Affiliations:** ^1^ Department of Clinical Pharmacology, School of Medicine, Muhimbili University of Health and Allied Sciences, Dar es Salaam, Tanzania; ^2^ Department of Global Health and Population, Harvard T.H. Chan School of Public Health, Boston, MA, United States; ^3^ Department of Pharmaceutical Sciences, School of Pharmacy, William Carey University, Hattiesburg, MS, United States; ^4^ Department of Clinical Pharmacy and Pharmacology, School of Pharmacy, Muhimbili University of Health and Allied Sciences, Dar es Salaam, Tanzania; ^5^ Department of Internal Medicine, School of Medicine, Addis Ababa University, Addis Ababa, Ethiopia; ^6^ Department of Pharmacology and Clinical Pharmacy, College of Health Sciences, Addis Ababa University, Addis Ababa, Ethiopia; ^7^ Center for Innovative Drug Development and Therapeutic Trials for Africa (CDT Africa), College of Health Sciences, Addis Ababa University, Addis Ababa, Ethiopia; ^8^ Department of Clinical Pharmacology and Pharmacoepidemiology, University of Heidelberg, Heidelberg, Germany; ^9^ Division of Clinical Pharmacology, Department of Laboratory Medicine, Karolinska Institutet, Stockholm, Sweden

**Keywords:** human immunodeficiency virus, tuberculosis, CYP2B6, Africa, pharmacaeconomics, population variation

## Abstract

Efavirenz-based combination antiretroviral-therapy (cART) is the recommended regimen during tuberculosis (TB) therapy. In a multi-national parallel prospective-cohort study, we investigated the impact of population and pharmacogenetic variations for efavirenz pharmacokinetics, auto-induction, and immunologic outcome during antituberculosis treatment. A total of 921 treatment-naïve HIV patients with (196 Ethiopians and 231 Tanzanians) or without TB co-infection (285 Ethiopians and 209 Tanzanians) were enrolled and treated with efavirenz-based cART. TB-HIV patients started rifampicin-based anti-TB therapy 4 weeks before cART. Efavirenz plasma concentrations were measured on the 4th and 16th weeks of cART. Genotyping for *CYP2B6*, *CYP3A5*, *ABCB1*, *UGT2B7*, and *SLCO1B1* was done. CD4 cells-count was measured at baseline, 12th, 24th, and 48th weeks of cART. Among HIV-only cohort, plasma efavirenz concentration and median CD4 cell count were significantly higher in Tanzanians than Ethiopians, and both *CYP2B6* genotype and population-variation were significant predictors of efavirenz plasma concentration. Within-population analyses indicated a pronounced efavirenz autoinduction in Tanzanians as reflected by a significant decrease of plasma efavirenz concentration over time (p = 0.0001), but not in Ethiopians. Among TB-HIV cohort, there were no significant between-population differences in plasma efavirenz concentrations or CD4 cell-recovery, and *CYP2B6* genotype but not population-variation was a significant predictor of efavirenz plasma exposure. In Tanzanian patients, short-term anti-TB co-treatment significantly reduced the mean plasma efavirenz concentration in CYP2B6*1/*1 genotype at week-4 (p = 0.005), but not at week-16 of cART. In Ethiopian patients, anti-TB cotreatment increased the mean plasma efavirenz concentration among CYP2B6*6 carriers at week-4 (p = 0.003) and week-16 (p = 0.035) of cART. In general, long-term anti-TB co-treatment increased plasma efavirenz concentration at week 16 of cART in both Ethiopians and Tanzanians being higher in CYP2B6*6/*6 > *1/*6 > *1/*1 genotypes. In TB-HIV patients, baseline body mass index (BMI), viral load, and WHO clinical-stage but not genotype, population-variation, or efavirenz concentration were significant predictors of immunologic outcome at week-48. In summary efavirenz auto-induction, pharmacokinetics, and the immunologic outcome are influenced by population-variation, anti-TB co-medication, and *CYP2B6* genotype. *CYP2B6* genotype is a significant predictor of efavirenz plasma exposure regardless of population-variation or antituberculosis co-treatment, but population-variation is insignificant during antituberculosis treatment. *CYP2B6* genotype, population, and geographic differences need to be considered for efavirenz dosage-optimization.

## Introduction

Tuberculosis (TB) continues to be the most common cause of mortality among patients infected with human immunodeficiency virus (HIV). The burden of this dual global epidemic of HIV and TB is extremely high in sub-Saharan Africa with the World Health Organization (WHO) estimating 86% of the deaths related to HIV associated TB coming from this region (WHO, 2017). Effective treatment for both TB and HIV diseases simultaneously poses numerous challenges because of potential interactions between antituberculosis and antiretroviral, clinical deterioration from immune reconstitution inflammatory syndrome, overlapping adverse events, and high medication burden (Tiberi et al., 2017).

Of importance in TB-HIV co-treatment are rifamycin derivatives, which induce the hepatic cytochrome P450 enzyme system resulting in increased metabolism and lowering serum concentrations of antiretrovirals, including efavirenz (Marzolini et al., 2001). Efavirenz based combination antiretroviral therapy (cART) is currently the treatment of choice for patients who are receiving rifampicin based antituberculosis co-treatment in most countries, including Ethiopia and Tanzania. As a result, there is a concern that the co-administration of rifampicin may increase the risk of virologic failure for HIV/TB patients receiving efavirenz-based regimens (Marzolini et al., 2001). Plasma efavirenz levels in the presence of rifampicin are highly variable between patients partly due to pharmacogenetic variations (Mukonzo et al., 2014b). Patients homozygous for the defective CYP2B6*6 variant allele, which is more common among African populations as compared to Caucasians, tend to experience higher levels of efavirenz when it is co-administered with rifampicin (Kwara et al., 2011).

Sub-Saharan African populations display the highest level of genetic and phenotypic diversity than any other race in the world (Gomez et al., 2014). Previous studies have shown higher levels of genetic diversity within black Africans compared to other non-Africans populations (Campbell and Tishkoff, 2008: Jakobsson et al., 2008: Dandara et al., 2014), and genetic diversity reduces with distance from East Africa (Prugnolle et al., 2005: Kanitz et al., 2018). Even within East Africa populations, there is wide genetic, environmental, cultural, and linguistic diversity. For instance, Ethiopians are predominantly of Semitic and Cushitic origin while Tanzanians comprise predominantly of Bantu and Nilotic origins. Apart from genetic and environmental factors, nutrition, geographical and population variation, and the use of traditional medicine may also contribute to between-patient and population variation, which may alter the extent of drug metabolism, efficacy, and adverse event profiles (Aklillu et al., 2002: Djordjevic et al., 2008: Hatta et al., 2015). For instance, the prevalence of efavirenz-based cART-associated liver and CNS toxicity profiles varies significantly between Ethiopians and Tanzanians (Yimer et al., 2006: Yimer et al., 2008: Mugusi et al., 2012: Mugusi et al., 2018).

The role of between population variations for efavirenz pharmacokinetics, auto-induction, and the immunological outcome is well investigated (Stohr et al., 2008: Ngaimisi et al., 2013). However, its impact during concomitant anti-tuberculosis regimen known to induce/inhibit the metabolism and cellular transport of antiretrovirals is not well understood. There are conflicting reports on the impact of pharmacogenetic diversity and population differences on the interaction between efavirenz and rifampicin from different populations, with some reporting decreased efavirenz plasma exposure by concomitant rifampicin co-treatment whereas others report no effect or increased plasma efavirenz concentration (Lopez-Cortes et al., 2002: Friedland et al., 2006: Gengiah et al., 2012: Habtewold et al., 2015).

Given the high genetic diversity and prevalence of TB-HIV coinfection in Sub-Saharan Africa, it is important to investigate the role of population differences including environmental and cultural diversity on antiretroviral and anti-TB drug interaction and the resulting impact on the treatment outcomes including safety and efficacy. Characterization of pharmacogenetics, pharmacokinetics, enzyme induction, and treatment outcomes between different African populations would form a base for personalized medicine and population-specific rationalized efavirenz dose adjustment strategies during anti-TB co-treatment in Africa. This study aimed at evaluating the impact of population and pharmacogenetic variation for efavirenz-rifampicin interaction, efavirenz pharmacokinetics, and immunologic outcome in Tanzanians and Ethiopians—the two east African populations that display wide genetic, environmental, cultural, nutritional, geographical, and linguistic variations.

## Patients and Methods

### Study Population and Setting

This study is part of a larger prospective cohort study funded by European and Developing Countries Clinical Trial Partnership (EDCTP) titled “Optimization of tuberculosis and HIV co-treatment in Africa: Pharmacokinetic and pharmacogenetic aspects on drug-drug interactions between rifampicin and efavirenz.” The study design, population eligibility criterions were described previously (Mugusi et al., 2012: Habtewold et al., 2015). In brief newly diagnosed TB patients with HIV co-infection who fulfilled inclusion criteria of age ≥18 years, CD_4_ count <200 cells/µl, naïve to cART were recruited and enrolled. Patients were started with a fixed drug combination consisting of rifampicin, isoniazid, ethambutol, and pyrazinamide for 2 months of intensive phase, followed by a continuation phase of 4 months with rifampicin and isoniazid for a total duration of 6 months. After 4 weeks of anti-TB treatment, cART was initiated with 2 nucleoside reverse-transcriptase inhibitors (NRTI) [either stavudine/lamivudine (d4T/3TC), zidovudine/lamivudine (AZT/3TC), or tenofovir/lamivudine (TDF/3TC)] and efavirenz and the patients were followed up to 1 year to monitor the immunological outcome. A parallel cohort of ART naïve HIV-only patients (209 Tanzanian and 285 Ethiopian) were enrolled as a control group and initiated efavirenz-based cART only and followed up for the same duration of time (Ngaimisi et al., 2013).

The study received ethical approval from Institutional Review Board (IRB) of the Muhimbili University of Health and Allied Sciences in Dar es Salaam, Tanzania, by the IRB of Faculty of Medicine, Addis Ababa University, and Ethiopian National Ethics Review Committee and by the IRB of Karolinska Institutet in Stockholm, Sweden. Prior written informed consent was obtained from all study participants.

### Clinical and Laboratory Parameters

At enrollment baseline, clinical and laboratory parameters were assessed. Pretreatment laboratory tests included complete and differential blood counts, hemoglobin, platelet count, CD_4_ cell count, HIV RNA determination, hepatitis B surface antigen, anti-hepatitis C antibody, serum albumin, renal function tests, and liver enzymes including serum aspartate aminotransferase (AST), alanine aminotransferase (ALT), alkaline phosphatase (ALP), and direct and total bilirubin levels. Change in CD_4_ count from baseline was monitored at 12^th^, 24^th^, and 48^th^ weeks of ART in both TB-HIV and HIV only cohorts.

### Efavirenz Plasma Concentration Determination

At 4 and 16 weeks of initiating efavirenz-based cART, a 16-h post-efavirenz dose blood samples were collected in vacutainer Cell Preparation Tubes (CPT) tubes (Becton Dickinson, Heidelberg, Germany). Blood samples were centrifuged (1,700 g for 20 min) to prepare plasma and stored at −80°C. Plasma efavirenz concentrations were quantified by liquid chromatography-tandem mass spectrometry (LC/MS/MS) as described previously (Ngaimisi et al., 2010). Efavirenz was quantified using ^13^C_6_-efavirenz as internal standards where the lower limits of quantification were 0.01 µg/ml. The efavirenz calibration range was 0.01–10 µg/ml. Linear regression with 1/x weighing resulted in correlation coefficients of r^2^ 0.99. Accuracy and precision (within-batch and batch-to-batch) of the assay fulfilled all recommendations of FDA guidelines.

### Genotyping for *CYP2B6*, *CYP3A5*, *ABCB1*, *SLCO1B1*, and *UGT2B7*

Genomic DNA was isolated from peripheral blood leukocytes using QIAamp DNA Maxi Kit (QIAGEN GmbH, Hilden, Germany). Genotyping for the common functional variant alleles in five relevant genes for efavirenz disposition were done using allelic discrimination TaqMan genotyping assays (Applied Biosystems, CA, USA) with the following ID number for each single nucleotide polymorphisms (SNP): (C:7586657_20 for *ABCB1 c.3435C > T* rs1045642, C:11711730_20 for CYP2B6*6 c.516G > T rs3745274, C:30720663_20 for UGT2B7 g.-327G > A rs7662029 (UGT2B7*2), C:26201809_30 for CYP3A5*3 c.6986A > G rs776746, C:30203950_10 for CYP3A5*6 14690G > A g.14690G > A, C:32287188_10 for CYP3A5*7 g.27131_27132insT rs241303343, C:_1901697_20 for *SLCO1B1* c.388A > G rs2306283 (*1b), and C:30633906_10 for *SLCO1B1 c.521T > C* rs4149056 (*5) on ABI 7500 FAST (Applied Biosystems, Foster City, CA). The final volume for each reaction was 10 μl, consisting of 2 X TaqMan Universal PCR Master Mix (Applied Biosystems), 20 X drug metabolizing genotype assay mix, and 10 ng genomic DNA. The PCR profile consisted of an initial step at 50°C for 2 min and 50 cycles with 95°C for 10 min and 92°C for 15 s. Genotyping for SLCO1B1 c.388A > G (rs2306283) and c.521T > C (rs4149056) in Tanzania was done using Light Cycler H based method (Aklillu et al., 2011; Aklillu et al., 2016).

### Statistical Analysis

Baseline socio-demographic and laboratory parameters were described as means and standard deviations (SD) or medians and interquartile range (IQR) for continuous variables and as percentages for categorical variables. The normality of kinetic data was assured by transforming the data to log 10 values before statistical analysis. Independent t-tests were used to compare log-transformed plasma efavirenz concentration at 4 and 16 weeks after cART initiation between Tanzanian and Ethiopian patients. Predictors of efavirenz plasma concentrations were assessed using univariate and multivariate regression analysis. Linear regression models were used to estimate predictors of log efavirenz concentrations at 4 and 16 weeks, separately. Similarly, the predictors of immunological outcomes at week 48 were assessed. Log-binomial regression analysis was used to estimate the relative risk of efavirenz concentrations <1 µg/ml at 4 and 16 weeks, separately. To take into account the repeated measure design and examine predictors of efavirenz longitudinally, we used generalized estimating equations (GEEs) with an exchangeable working covariance and identify the link to produce population-averaged mean differences in efavirenz concentrations while the log link for the population-averaged relative risk of efavirenz <1 µg/ml. Variables in univariate analysis with a *p <*0.2 were included in all multivariate analyses. All *p-*values were two-sided, and *p-*values of < 0.05 were considered statistically significant. Statistical analyses were performed using Stata version 15 (StataCorp. 2017. Stata Statistical Software: Release 15. College Station, TX: StataCorp LLC).

## Results

### Study Population and Baseline Characteristics

A total of 921 treatment naïve HIV patients with or without TB-HIV coinfection were enrolled in a multi-national parallel prospective cohort study: 427 TB-HIV co-infected patients (196 in Ethiopia and 231 in Tanzania) and 494 HIV patients without TB coinfection (285 in Ethiopia and 209 in Tanzania). The HIV only cohort treated with efavirenz-based cART only were used as a control group to examine the impact of population differences on efavirenz-rifampicin drug interaction. The sociodemographic, clinical, and laboratory, pharmacogenetic, and pharmacokinetic data of the HIV-only cohort is described previously (Ngaimisi et al., 2013).

The sociodemographic, baseline clinical, and laboratory characteristics of the TB-HIV cohort are presented in **Table 1**. Sputum smear microscopy for tuberculosis showed that 43.8% were smear-positive, and the remaining were diagnosed to have smear negative-pulmonary tuberculosis (**Table 1**). The mean (±SD) BMI of all patients was 19.2 ± 3.2 but Ethiopians had a significantly higher proportion of patients with a BMI ≤18.5 kg/m^2^ compared to the Tanzanians (49.5 *vs*. 39.3% respectively) (p = 0.03). The mean (±SD) hemoglobin was 10.6 ± 2.2 with Tanzanians (19%) having significantly more patients with a hemoglobin of <8.5g/dl compared to the Ethiopians (10%) (p = 0.02). The patients were severely immunocompromised with a mean CD_4_ cell count of 97.7 cells/µl with over half having a CD_4_ cell count of <100 cells/µl in both settings.

**Table 1:** Sociodemographic and baseline clinical characteristics of Tanzanian and Ethiopian TB-HIV patients on efavirenz-based combination antiretroviral-therapy (cART) and rifampicin based anti-TB therapy.

Variable		Tanzania N = 231	Ethiopia N = 196
Sex	Males	117 (50.6%)	103 (52.6%)
	Females	114 (49.4%)	93 (47.4%)
Median age in years (IQR)		38 (12)	35 (13)
Marital status	Single	61 (26.4%)	69 (35.2%)
	Married	116 (50.2%)	68 (34.7%)
	Widowed	20 (8.7%)	27 (13.8%)
	Divorced	34 (14.7%)	32 (16.3%)
Education	Illiterate	9 (3.9%)	28 (14.3%)
	Able to read/write and primary	185 (80.1%)	88 (44.9%)
	Secondary and tertiary	37 (16.0%)	80 (40.8%)
Type of TB	Smear positive	155 (67.1%)	32 (16.3%)
	Smear negative	76 (32.9%)	164 (83.7%)
WHO stages	Stage III	217 (93.9%)	130 (66.3%)
	Stage IV	14 (6.1%)	66 (33.7%)
cART	d4T+3TC+EFV	116 (50.9%)	85 (45.4%)
	AZT+3TC+EFV	112 (49.1%)	48 (25.7%)
	TDF+3TC+EFV	0	54 (28.9%)
BMI (Kg/m^2^) [median, IQR]		19.3 [3.7]	18.6 [2.8]
BMI categories	<18.5	90 (39.3%)	97 (49.5%)
	>18.5	139 (60.7%)	99 (50.5%)
Hb (g/dl) [median, IQR]		9.7 [2.3]	11.3 [3.2]
Severe anemia (< 8.5g/dl)	Yes	39 (18.8%)	20 (10.2%)
	No	169 (81.2%)	176 (89.8%)
AST (IU/L) [median, IQR]		28.7 [24.2]	43 [37.5]
ALT (IU/L) [median, IQR]		17 [18.6]	29 [20.5]
Hepatitis B	Negative	163 (96.4%)	181 (92.3%)
	Positive	6 (3.6%)	15 (7.7%)
Hepatitis C	Negative	182 (98.9%)	191 (97.9%)
	Positive	2 (1.1%)	4 (2.1%)
CD4 (cells/µl) [median, IQR]		94 [122]	78.5 [87.5]
CD4 categories	<100 cells/µl	118 (51.1%)	113 (57.7%)
	100–200 cells/µl	113 (48.9%)	83 (42.3%)
Viral load (copies/ml) [median, IQR]		226,000 [542,200]	169,758 [330,568]

WHO stage, World Health Organization clinical staging; cART, combination antiretroviral therapy; d4T, stavudine; AZT, zidovudine; TDF, tenofovir; 3TC, lamivudine; EFV, efavirenz; BMI, body mass index; Hb, hemoglobin; AST, aspartate aminotransferase; ALT, alanine aminotransferase.

### Distribution of Allele and Genotype Frequencies in Ethiopians and Tanzanians

Three hundred and twenty-eight TB-HIV coinfected patients had their genotype determined (145 Tanzanians and 183 Ethiopians). Comparison of *CYP2B6, CYP3A5, UGT2B7, ABCB1,* and *SLCO1B1* genotype and variant allele frequency distribution between Ethiopians and Tanzanian TB-HIV patients is presented in **Table 2**. Except for CYP2B6*6 and CYP3A5*6, the distribution of all other variant alleles genotypes for were significantly different between the two study populations. Though not significant, Tanzanians had a higher allele frequency of the defective CYP2B6*6 variant allele than Ethiopians. Proportionately more Tanzanians had the *6/*6 allele compared to the Ethiopians (58.8% compared to 41.2%). The defective variant allele CYP3A5*7 was absent in Ethiopians but occurred at a frequency of 10% in Tanzanians. Due to the high frequency (70%) of the defective CYP3A5*3 variant allele, the frequency of functional CYP3A5*1 allele in Ethiopians was significantly lower in Ethiopians (22.3%) than Tanzanians (50.4%). Likewise, the defective SLCO1B1*5 variant allele occurs at a much higher frequency in Ethiopians than Tanzanians.

**Table 2:** Comparison of *CYP2B6*, *CYP3A5*, *UGT2B7*, *ABCB1*, and *SLCO1B1*genotype and allele frequencies distribution of functional variant alleles among TB-HIV co-infected Ethiopians and Tanzanians patients.

Genotype		Ethiopia	Tanzania	p
		N (%)	N (%)	
CYP2B6*6	*1/*1	74 (50.0%)	72 (49.7%)	0.25
	*1/*6	62 (41.9%)	53 (36.6%)	
	*6/*6	12 (8.1%)	20 (13.8%)	
CYP3A5*3	*1/*1	28 (15.3%)	87 (60.0%)	<0.001
	*1/*3	87 (47.5%)	51 (35.2%)	
	*3/*3	68 (37.2%)	7 (4.8%)	
CYP3A5*6	*1/*1	127 (69.4%)	102 (70.3%)	0.85
	*1/*6	49 (26.8%)	36 (24.8%)	
	*6/*6	7 (3.8%)	7 (4.8%)	
CYP3A5*7	*1/*1	183 (100%)	116 (80.0%)	<0.001
	*1/*7	0	29 (20.0%)	
Number of CYP3A5*1 allele	Zero	111 (60.7%)	41 (28.3%)	<0.0001
	One	62 (33.9%)	62 (42.8%)	
	Two	10 (5.5%)	42 (29.0%)	
UGT2B7*2	*AA*	45 (24.6%)	15 (10.3%)	<0.0001
	*AG*	90 (49.2%)	61 (42.1%)	
	*GG*	48 (26.2%)	69 (47.6%)	
*ABCB1 c.3435C > T*	*CC*	118 (64.5%)	109 (75.2%)	0.10
	*CT*	54 (29.5%)	31 (21.4%)	
	*TT*	11 (6.0%)	5 (3.4%)	
*ABCB1 c.4036A > G*	*AA*	126 (68.9%)	72 (49.7%)	0.0007
	*AG*	53 (29.0%)	62 (42.8%)	
	*GG*	4 (2.2%)	11 (7.6%)	
SLCO1B1 D130N (*1b)	*AA*	24 (13.1%)	3 (2.1%)	<0.0001
	*AG*	102 (55.7%)	32 (22.2%)	
	*GG*	57 (31.1%)	109 (75.7%)	
SLCO1B1 A174V (*5)	*TT*	112 (61.2%)	133 (92.4%)	<0.0001
	*CT*	67 (36.6%)	11 (7.6%)	
	*CC*	4 (2.2%)	0 (0.0%)	
**Allele frequency**				
CYP2B6*6		29.1%	32.1%	0.42
CYP3A5*3		60.9%	22.4%	<0.0001
CYP3A5*6		17.2%	17.3%	0.99
CYP3A5*7		0	11.1%	<0.0001
Number of CYP3A5*1 allele		22.4%	50.3%	<0.0001
UGT2B7*2		50.8%	68.6%	<0.0001
*ABCB1 c.3435C > T*		20.8%	14.1%	0.02
*ABCB1 c.4036A > G*		16.7%	30.0%	0.0001
SLCO1B1 D130N (*1b)		59.0%	86.8%	<0.0001
SLCO1B1 A174V (*5)		20.0%	3.8%	<0.0001

### Efavirenz Plasma Concentrations On and Off Rifampicin Co-Treatment

**Figure 1:**
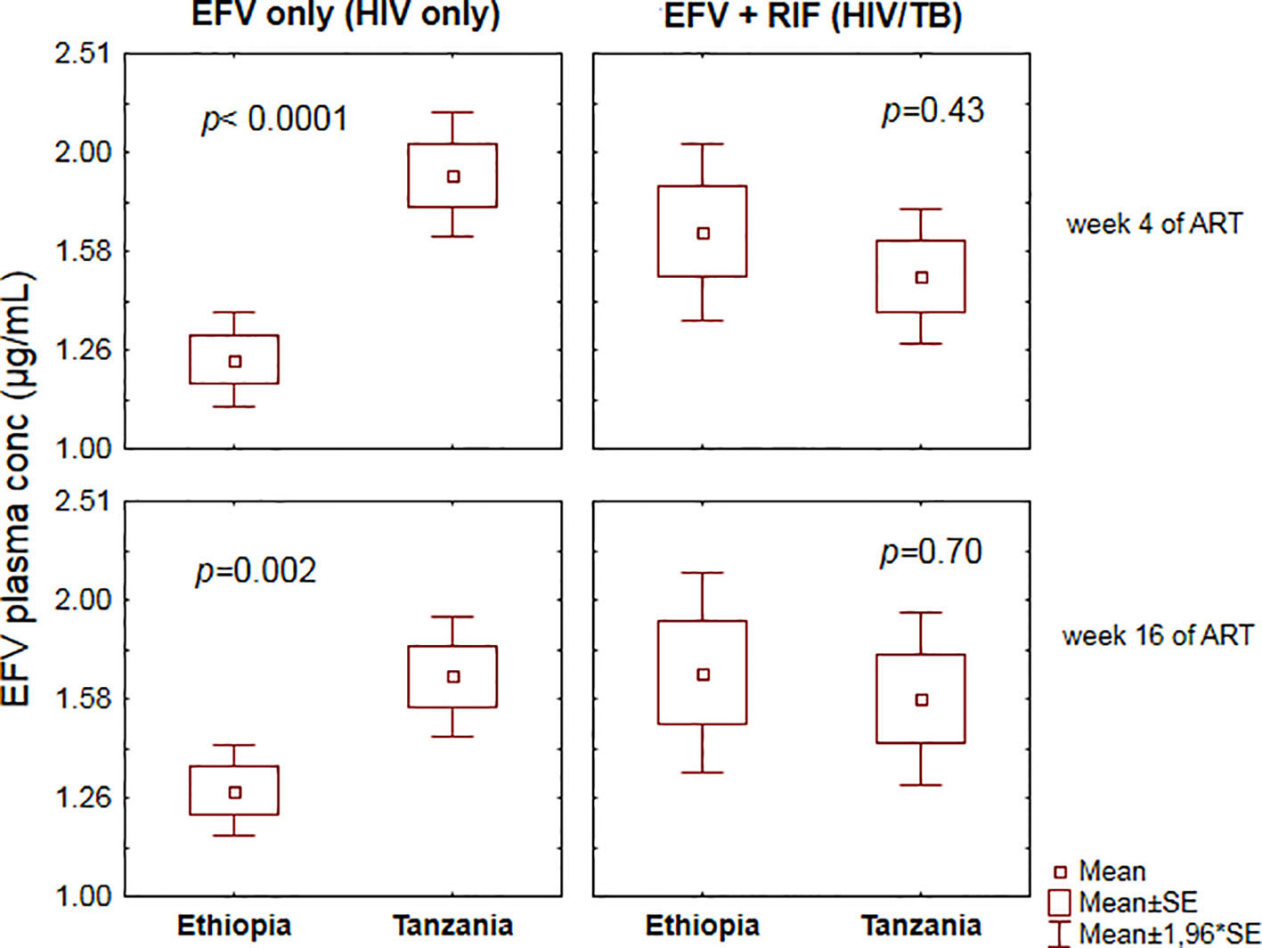
Comparison of mean ± standard errors efavirenz plasma concentration between Ethiopian and Tanzanian patients at the 4^th^ and 16^th^ weeks of efavirenz-based *combination antiretroviral-therapy* (cART) using independent t-test.

Comparison of plasma efavirenz concentration between Ethiopian and Tanzanian HIV patients receiving efavirenz-based cART only (control group) *versus* TB-HIV patients co-treated with efavirenz (EFV) based cART and rifampicin based anti-TB therapy at the 4^th^ and 16^th^ week of ART is presented in **Figure 1**. In the HIV only cohort, there were significant differences in efavirenz plasma concentration between the two study populations, being higher in Tanzanian than Ethiopian patients both at the 4^th^ and 16^th^ weeks of cART (**Figure 1**). However, in TB-HIV patients co-treated with rifampicin based anti-TB therapy, there was no significant between-population difference in plasma efavirenz exposure observed at both study time points. Though not significant, the geometric mean (95% confidence interval) of efavirenz plasma concentration in Tanzanian TB-HIV patients was slightly lower compared to the Ethiopian patients [1.49 (95% CI: 1.27–1.76) µg/ml and 1.66 (95% CI: 1.35–2.05) µg/ml, respectively].

**Figure 2:**
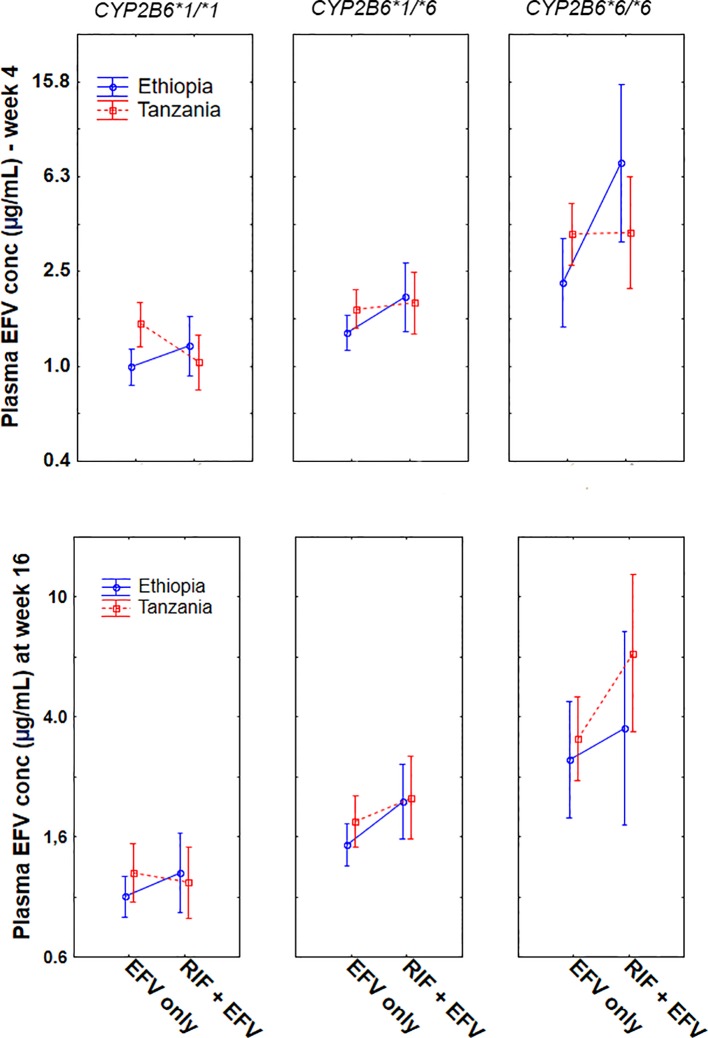
Comparison of mean log efavirenz plasma concentrations in the absence [efavirenz (EFV) only] and presence of rifampicin based anti-TB cotreatment [EFV + (rifampicin) RIF] among Ethiopian and Tanzanian HIV patients stratified by CYP2B6*6 genotype at week 4 and week 16 of *combination antiretroviral-therapy (*cART). Bars indicate mean ± 95% confidence intervals of the mean.

Comparison of plasma efavirenz concentration while on (EFV+RIF) and off (EFV-only) rifampicin co-treatment among Ethiopians and Tanzanians stratified by CYP2B6*6 genotype group at week 4 and 16 of cART is presented in **Figure 2**. In Tanzanian patients, short term rifampicin co-treatment significantly reduced plasma efavirenz concentration (week-4 of cART), particularly in CYP2B6*1/*1 genotype group (p = 0.005), but no significant effect was observed in Tanzanian CYP2B6*6 carriers. On the other hand, though not significant, there was a trend of having higher mean efavirenz plasma concentration among those co-treated with rifampicin than those without rifampicin in Tanzanian CYP2B6*6 carriers (p = 0.18) at week 16 of cART but not in CYP2B6*1/*1 (p = 0.68).

In contrast, among Ethiopian patients, anti-TB cotreatment increased the mean plasma efavirenz concentration, particularly among carriers of CYP2B6*6 allele both at week 4 (p = 0.003) and at week 16 (p = 0.035). Though not significant, a similar trend was observed in CYP2B6*1/*1 genotype at week 4 (p = 0.18) and at week 16 (p = 0.36). Overall long-term rifampicin co-treatment increased plasma efavirenz concentration in Ethiopians being higher in CYP2B6*6/*6 > *1/*6 > *1/*1 (**Figure 2**).

### Change in Plasma Efavirenz Concentration Overtime

**Figure 3:**
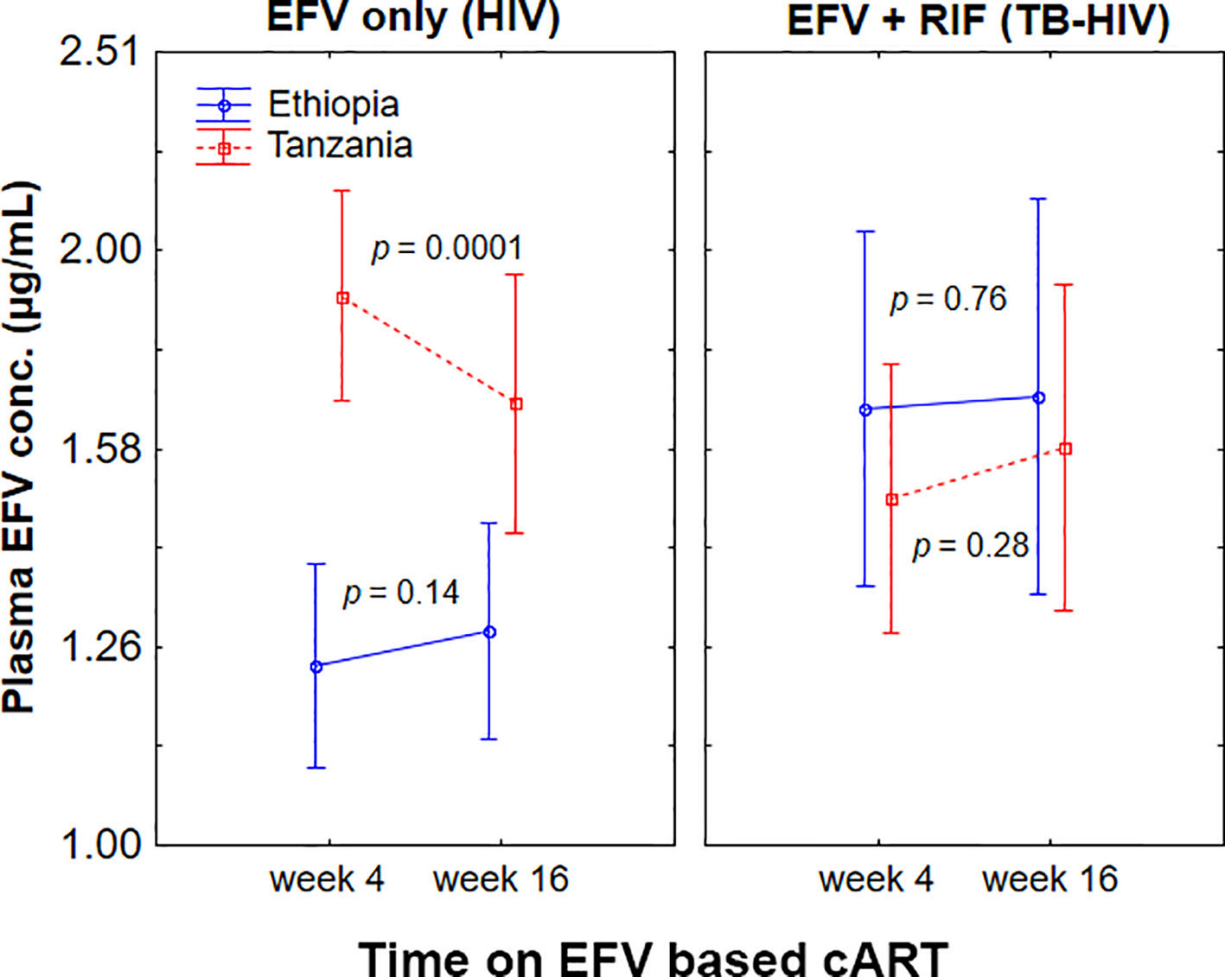
Comparison of change in plasma efavirenz concentration from week 4 to week 16 of *combination antiretroviral-therapy* (cART) among Ethiopian and Tanzanian HIV patients treated with efavirenz based cART only [efavirenz (EFV) only] or co-treated with rifampicin based antituberculosis therapy [EFV + rifampicin (RIF)]. Bars indicate mean ± 95% confidence intervals of the mean.

Change in plasma efavirenz concentration from week 4 to 16 of cART stratified by treatment group and study population is presented in **Figure 3**. Repeated measure ANOVA indicated a significant interaction between country and treatment arm in influencing plasma efavirenz concentration over time (p = 0.006). Among HIV patients treated with efavirenz-based cART only, within-population analyses using paired samples t-test indicated a pronounced efavirenz autoinduction in Tanzanians as reflected by a significant decrease in the mean plasma efavirenz concentration at week 16 than week 4 (p = 0.0001), whereas no significant differences in plasma concentration over time were observed in Ethiopians (p = 0.14). On the other hand, no significant change in efavirenz plasma concentration between the 4^th^ and 16^th^ weeks of cART was observed in TB-HIV patients co-treated with rifampicin based anti-TB therapy regardless of country of origin (**Figure 2**).

### Proportion of Patients in the Different Efavirenz Therapeutic Range

Previous studies have suggested that plasma efavirenz concentration below 1 and above 4 µg/ml predict treatment failure and central nervous toxicity respectively (Marzolini et al., 2001). Comparison of the proportion of patients in the different plasma efavirenz therapeutic ranges (< 1, 1–4, and > 4 µg/ml) at week 4 and week 16 among patients treated with efavirenz-based cART without (EFV only) or with rifampicin based anti-TB (EFV + RIF) stratified by country is presented in **Table 3**. In EFV only group, the proportion of patients having plasma efavirenz concentration below the proposed therapeutic range (<1 µg/ml) was two-fold higher in Ethiopians than in Tanzanian counterparts, while proportion of patients having plasma concentration above the therapeutic range (>4 µg/ml) was three-fold higher in Tanzanians than Ethiopians. In contrast, no country-specific difference in the proportion of patients in the different therapeutic ranges was observed among TB-HIV cohort co-treated with rifampicin based anti-tuberculosis therapy. Nearly a third of the patients (31%) had sub-therapeutic efavirenz plasma concentrations, with only 53% being within the recommended therapeutic range at weeks 4 or 16 after cART initiation.

**Table 3:** The proportion of patients in the various plasma efavirenz concentration therapeutic range stratified by country and treatment group.

Treatment group	**Country**	**Week 4 of cART**				**Week 16 of cART**			
		**<1 µg/L**	**1 to 4 µg/L**	**>4 µg/L**	**p**	**<1 µg/L**	**1 to 4 µg/L**	**>4 µg/L**	**p**
**EFV only**	Ethiopia	74	129	12	<0.001	62	120	9	0.0005
		34.42%	60.00%	5.58%		32.46%	62.83%	4.71%	
	Tanzania	32	120	53		26	87	21	
		15.61%	58.54%	25.85%		19.40%	64.93%	15.67%	
**EFV + RIF**	Ethiopia	20	39	12	0.89	21	26	9	0.91
		28.17%	54.93%	16.90%		37.50%	46.43%	16.07%	
	Tanzania	34	71	18		29	41	15	
		27.64%	57.72%	14.63%		34.12%	48.24%	17.65%	

### Factors Influencing Plasma Efavirenz Concentration on and Off Rifampicin Co-Treatment

Predictors of efavirenz plasma concentration in HIV patients without TB coinfection is presented previously (Ngaimisi et al., 2013). In brief geographic differences (patient country), CYP2B6*6 and *ABCB1 c.4036A/G (rs3842)* genotypes were significant predictors of efavirenz plasma concentrations.

Predictors of plasma efavirenz concentrations in TB-HIV patients co-treated with rifampicin based anti-TB therapy is presented in **Table 4**. Country was not associated with efavirenz concentrations at 4 or 16 weeks (p-values > 0.05). In the multivariate model at 16 weeks of cART, there was some indication that males had lower efavirenz concentrations (mean difference log efavirenz: −0.24; 95% CI: −0.49–0.02; *p* = 0.07), but results did not reach statistical significance. *CYP2B6* genotype was a significant predictor of efavirenz concentrations at both 4 and 16 weeks. In the longitudinal model, patients with CYP2B6*6/*6 and *1/*6 alleles had 1.38 (95% CI: 0.98–1.78) and 0.52 (95% CI: 0.28–0.77) greater log efavirenz plasma concentrations than patients who had CYP2B6 *1/*1 genotype. Predictors for efavirenz plasma concentrations below the therapeutic window threshold (<1 µg/ml) were also assessed. Statistically significant differences were seen in the *CYP2B6* genotype distribution and efavirenz levels where 71% of the patients with <1 µg/ml were CYP2B6 *1/*1 and 48% of patients with supra-therapeutic efavirenz concentrations (> 4 µg/ml) were *6/*6 (*p* < 0.001). Multivariate analysis showed that patients with CYP2B6 *1/*6 and *6*6 genotype had 0.50 (95% CI: 0.29–0.87) and 0.23 (95% CI: 0.05–1.03) times the risk of having efavirenz concentrations <1 µg/ml as compared to those with *1/*1 genotype respectively (**Table 4**). A total of 44% of the patients had both the CYP2B6 *1/*1 and no CYP3A5*1 allele, and these patients had a 2.20 (95% CI: 1.25–3.82) times higher risk of having efavirenz concentrations <1 µg/ml compared to those who did not have both alleles.

**Table 4:** Univariate and multivariate regression analysis of plasma efavirenz concentration at 4 weeks and 16 weeks of cART during rifampicin based anti-TB co-treatment.

Variable	Week 4				Week 16				Longitudinal GEE analysis of 4 and 16 weeks			
	Univariate analysis		Multivariate analysis		Univariate analysis		Multivariate analysis		Univariate analysis		Multivariate analysis	
	Mean diff (95% CI)	p-value	Mean diff (95% CI)	p-value	Mean diff (95% CI)	p-value	Mean diff (95% CI)	p-value	Mean diff (95% CI)	p-value	Mean diff (95% CI)	p-value
**Country**												
Tanzania	Ref		Ref		Ref				Ref		Ref	
Ethiopia	0.01 (−0.09–0.12)	0.79	−0.00 (−0.12–0.11)	0.98	0.01 (−0.12–0.14)	0.88	0.01 (−0.12–0.14)	0.92	0.03 (−0.07–0.13)	0.56	0.05 (−0.07–0.17)	0.45
**Sex**												
Females	Ref		Ref		Ref				Ref		Ref	
Males	−0.09 (−0.19–0.01)	0.07	−0.03 (−0.12–0.05)	0.43	−0.16 (−0.28–0.03)	0.013	−0.10 (−0.21–0.01)	0.07	−0.10 (−0.19–0.00)	0.046	−0.06 (−0.15–0.03)	0.17
**Type of TB**												
Smear positive	Ref		Ref		Ref				Ref		Ref	
Smear negative	0.07 (−0.03–0.17)	0.18	0.05 (−0.04–0.15)	0.27	−0.00 (−0.13–0.13)	0.97			0.08 (−0.02–0.18)	0.12	0.06 (−0.04–0.16)	0.21
**WHO stage**												
Stage III	Ref				Ref				Ref			
Stage IV	0.01 (−0.12–0.13)	0.90			0.02 (−0.12–0.18)	0.77			0.02 (−0.11–0.14)	0.76		
**HAART initiated**												
Stavudine	Ref				Ref				Ref			
Zidovudine	−0.02 (−0.12–0.09)	0.77			−0.05 (−0.18–0.09)	0.49			−0.06 (−0.16–0.04)	0.27		
Tenofovir	0.12 (−0.09–0.33)	0.28			0.12 (−0.17–0.40)	0.41			0.07 (−0.15–0.28)	0.54		
**BMI (kg/m^2^)**	0.00 (−0.01–0.02)	0.57			−0.00 (−0.02–0.02)	0.99			0.00 (−0.01–0.02)	0.94		
**Anemia**												
≥8.5g/dl	Ref				Ref				Ref			
<8.5g/dl	0.01 (−0.13–0.15)	0.86			−0.04 (−0.21–0.13)	0.65			0.04 (−0.09–0.18)	0.53		
**Hepatitis B**												
Negative	Ref				Ref				Ref			
Positive	−0.03 (−0.29–0.23)	0.81			0.19 (−0.09–0.48)	0.18	0.18 (−0.06–0.42)	0.13	0.12 (−0.12–0.36)	0.33		
**Hepatitis C**												
Negative	Ref				Ref				Ref			
Positive	0.17 (−0.23–0.58)	0.40			0.11 (−0.67–0.88)	0.78			0.22 (−0.21–0.66)	0.32		
**CD4 cell count**)												
<100 cells/µl	Ref		Ref		Ref				Ref		Ref	
≥100 cells/µl	0.11 (0.01–0.20)	0.037	0.08 (−0.00–0.17)	0.050	0.06 (−0.07–0.19)	0.34			0.09 (0.01–0.19)	0.07	0.08 (−0.01–0.17)	0.06
**VL log copies/ml**	−0.00 (−0.04–0.03)	0.89			0.01 (−0.03–0.05)	0.64			−0.00 (−0.03–0.03)	0.97		
***CYP2B6***												
*1/*1	Ref		Ref		Ref				Ref		Ref	
*1/*6	0.17 (0.08–0.27)	0.001	0.16 (0.06–0.26)	0.002	0.22 (0.10–0.35)	<0.001	0.22 (0.09–0.35)	0.001	0.25 (0.15–0.36)	<0.001	0.23 (0.12–0.33)	<0.001
*6/*6	0.70 (0.54–0.86)	<0.001	0.68 (0.51–0.84)	<0.001	0.71 (0.49–0.93)	<0.001	0.68 (0.46–0.91)	<0.001	0.63 (0.46–0.81)	<0.001	0.59 (0.42–0.77)	<0.001
Number of CYP3A5*1 allele												
Zero	Ref		Ref		Ref				Ref		Ref	
one	0.05 (−0.08–0.18)	0.46	−0.01 (−0.12–0.10)	0.88	−0.01 (−0.17–0.16)	0.93			0.08 (−0.05–0.20)	0.22	0.04 (−0.07–0.15)	0.49
Two	0.10 (−0.05–0.25)	0.18	0.02 (−0.13–0.16)	0.83	0.09 (−0.09–0.29)	0.31			0.17 (0.03–0.31)	0.021	0.10 (−0.04–0.25)	0.15
***ABCB1 c 3435 C/T***												
CC	Ref				Ref				Ref			
CT&TT	0.04 (−0.08–0.17)	0.51			−0.01 (−0.17–0.15)	0.91			−0.00 (−0.13–0.12)	0.97		
***SLCO1B1 A174V***												
TT	Ref				Ref				Ref			
CT&CC	0.04 (−0.09–0.18)	0.56			−0.12 (−0.29–0.06)	0.19	−0.03 (−0.19–0.13)	0.67	−0.02 (−0.16–0.12)	0.81		
***UGT2B7***												
AA	Ref				Ref				Ref			
AG&GG	0.02 (−0.14–0.17)	0.81			0.02 (−0.16–0.21)	0.79			0.03 (−0.13–0.18)	0.73		

cART, combination antiretroviral therapy; GEE, generalized estimating equation; WHO stage, World Health Organization clinical staging; BMI, body mass index.

### Predictors of Immunological Outcomes During Rifampicin Co-Treatment

**Figure 4:**
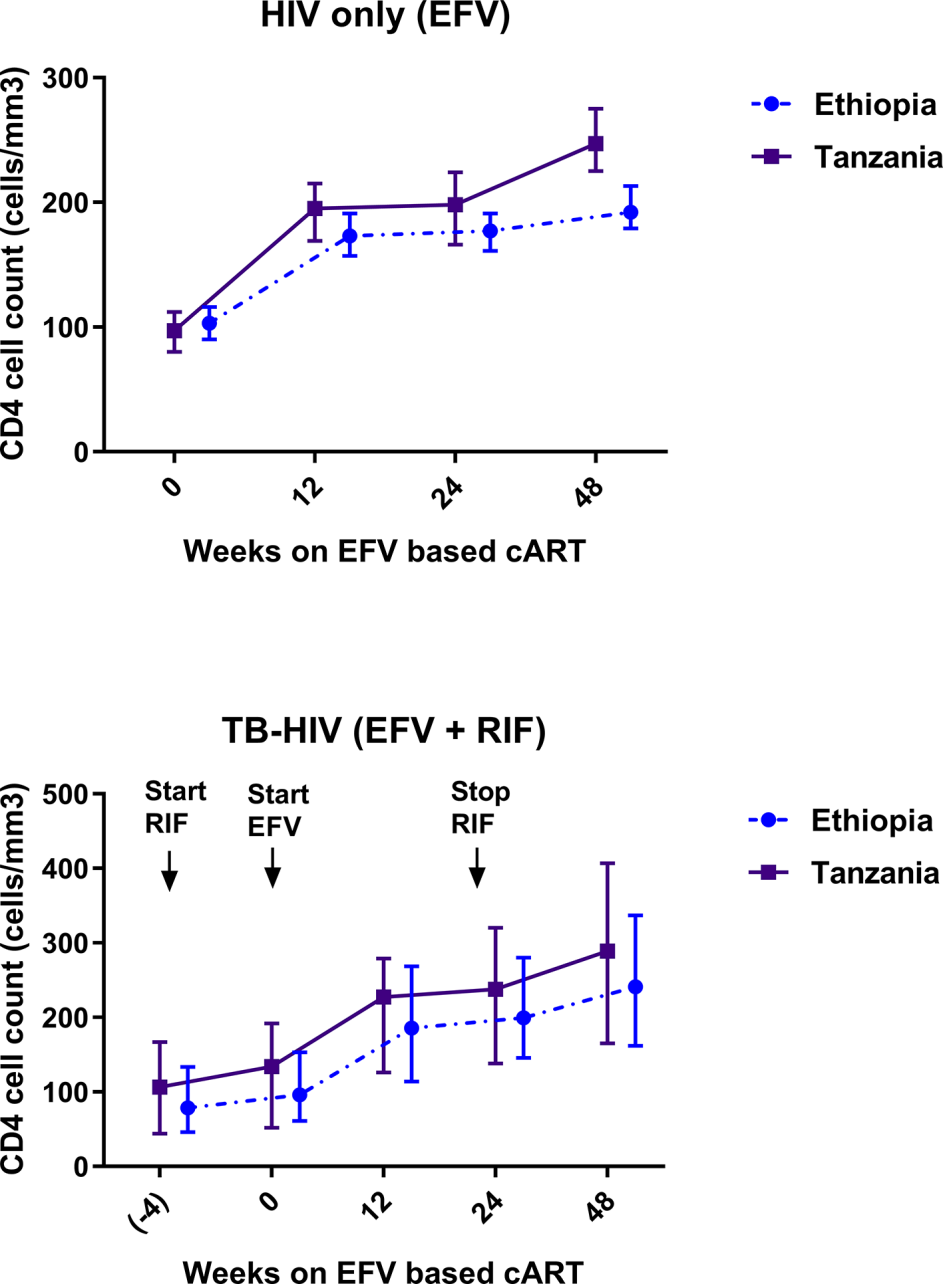
Comparison of change in median and interquartile range of CD4 cell count profile during combination antiretroviral-therapy (cART) among Ethiopian and Tanzanian HIV patients in the absence [efavirenz (EFV) only] and presence of anti-TB cotreatment [EFV + *rifampicin* (RIF)].

Comparison of the median and interquartile range (IQR) of CD_4_ cell count at baseline, 12^th^, 24^th^, and 48^th^ week of cART between Ethiopians and Tanzanians stratified by treatment group are presented in **Figure 4**. Among HIV only cohort treated with EFV based cART only, the median CD_4_ cell count was significantly higher in Tanzanians than Ethiopians at week 24 (p = 0.01) and week 48 (p = 0.001) of cART (p < 0.001). Likewise, and the percent change of CD_4_ cell count from baseline by week-24 (p = 0.03) and 48 (p = 0.02) were higher in Tanzanian than Ethiopians.

In contrast, no significant between-country differences in median CD_4_ cell count at week 24 (p = 0.82), week 48 (p = 0.19), or percent change in CD_4_ cell count from baseline by week 24 or 48 of cART was observed among TB-HIV patients co-treated with rifampicin based anti-tuberculosis. Though not significant the CD_4_ cell-count remained consistently higher in Tanzanians than Ethiopians (**Figure 4**). There was a gradual mean increase in the CD_4_ cell count from 98 (±63) cells/µl at baseline, to 231 cells/µl at 24 weeks, and 271 cells/µl at 48 weeks after cART initiation. Tanzanian patients had an overall higher median CD_4_ cell count compared to Ethiopians at week 24 (207 *vs*. 200 cells/µl) (*p =* 0.38) and at week 48 post cART (271 *vs*. 241 cells/µl) (*p =* 0.057). At the 48 weeks of cART, twice as many Ethiopians compared to Tanzanians had CD_4_ cell counts below 200 cells/µl (66% *vs*. 34% respectively).

Predictors of CD_4_ cell count by week 48 is presented in **Table 5**. Among TB-HIV patients, baseline BMI, viral load, and WHO clinical stage but not genotype, population variation, or plasma efavirenz concentration were significant predictors of the immunologic outcome by week 48.

**Table 5:** Univariate and multivariate correlates of CD4 percent change from baseline to 48 weeks of cART treatment among TB-HIV coinfected patients.

Variable	Univariate analysis		Multivariate analysis	
	Mean diff (95% CI)	p-value	Mean diff (95% CI)	p-value
**Country**				
Tanzania	Ref		Ref	
Ethiopia	1.72 (−48.9–52.3)	0.95	1.5 (−60.8–63.9)	0.96
**Sex**				
Female	Ref		Ref	
Male	−24.2 (−73.4–25.1)	0.33	−28.6 (−75.6–18.4)	0.23
**Type of TB**				
Smear positive	Ref			
Smear negative	35.9 (−14.9–86.8)	0.17	60.8 (−0.26–122.0)	0.051
**WHO stage**				
Stage III	Ref		Ref	
Stage IV	−60.4 (−117.2–3.59)	0.037	−101.0 (−161.6 – −40.5)	0.001
**cART initiated**				
Stavudine	Ref			
Zidovudine	−21.2 (−77.5–35.0)	0.46		
Tenofovir	−5.65 (−76.1–64.8)	0.87		
**BMI (kg/m^2^)**	−8.3 (−16.2–0.42)	0.039	−9.4 (−17.1 – −1.7)	0.017
**Anemia**				
≥8.5 g/dl	Ref		Ref	
<8.5 g/dl	99.4 (29.1–169.6)	0.006	60.7 (−10.8–132.2)	0.09
**Hepatitis B**				
Negative	Ref		Ref	
Positive	−99.5 (−220.1–21.2)	0.11	−98.7 (−212.4–14.9)	0.09
**Hepatitis C**				
Negative	Ref			
Positive	−30.0 (−284.5–224.5)	0.82		
**Log viral load copies/ml**	17.7 (2.5–32.9)	0.023	15.0 (0.67–29.4)	0.040
***CYP2B6***				
*1/*1	Ref			
*1/*6	6.9 (−51.2–65.1)	0.81		
*6/*6	−50.8 (−147.6–45.9)	0.30		
Number of CYP3A5*1 allele				
Zero	Ref			
One	−18.2 (−78.3–41.9)	0.55		
Two	−13.0 (−98.4–72.3)	0.76		
***ABCB1 c 3435 C/T***				
CC	Ref			
CT&TT	−20.0 (−79.8–39.7)	0.51		
***SLCO1B1 A174V***				
TT	Ref			
CT&CC	−24.9 (−84.1–34.2)	0.41		
***UGT2B7*** (g.−327G > A)				
AA	Ref			
AG&GG	−31.3 (−101.3–38.8)	0.38		
Log plasma efavirenz conc at week 4 of cART	7.8 (−75.9–91.6)	0.85		
Log plasma efavirenz conc at week 16 of cART	−32.0 (−123.1–59.0)	0.49		

## Discussion

In the present study we performed a parallel comparative multicenter prospective observational study to assess the importance of population, geographic, and pharmacogenetic variations for efavirenz pharmacokinetics and immunological outcome during antituberculosis cotreatment in two genetically different east African populations, Ethiopians, and Tanzanians. HIV patients without tuberculosis co-infection were enrolled as a control group to investigate the impact of population and geographic differences in the absence of anti-TB co-medication. Our result indicates different efavirenz plasma exposure and immunologic outcome profiles between Ethiopians and Tanzanian HIV patients depending on the presence or absence of antituberculosis co-medication. Tanzanian HIV patients treated with efavirenz-based cART only had significantly higher plasma efavirenz concentration and immunologic recovery than Ethiopian counterparts. However, this difference became insignificant during rifampicin based anti-TB co-medication. While the impact of *CYP2B6* genotype for efavirenz plasma exposure was prominent regardless of population variation or rifampicin co-medication, the importance of population variation was prominent only when efavirenz-based cART was given alone. This may indicate that findings during rifampicin based anti-TB co-treatment could possibly be extrapolated between black African populations, but not when efavirenz-based cART is given alone. To the best of our knowledge, this is the first parallel prospective study to explore the importance of between population variation for antiretroviral and antituberculosis drug interaction and its impact on efavirenz pharmacokinetics, auto-induction, and immunologic outcome in two genetically different sub-Saharan Africa population.

Change in plasma efavirenz concentration was determined at two different time points to investigate the short and long-term efavirenz auto-induction in the presence or absence of rifampicin co-treatment. Efavirenz induces its own metabolism *via* CYP2B6 and CYP3A4/5 enzymes (Ngaimisi et al., 2010: Habtewold et al., 2013). Our result indicates that the extent of efavirenz auto-induction is influenced by population differences, *CYP2B*6 genotype, and anti-tuberculosis co-treatment. Interestingly in the absence of anti-TB medication, efavirenz plasma concentration significantly reduced overtime particularly in Tanzanians than Ethiopians (**Figure 3**). The impact of rifampicin cotreatment in reducing efavirenz plasma concentration was prominent in CYP2B6*1/*1 genotype but not in CYP2B6*6 carriers, as revealed by a significant decline in efavirenz plasma concentration at week 4 of cART in Tanzanian CYP2B6*1/*1 genotype (**Figure 2**). This may indicate genotype-based preferential enzyme induction of the *CYP2B6* wild-type allele, or CYP2B6*6 could equally be induced, but since it codes for reduced enzyme activity no significant impact on plasma efavirenz concentration could be observed among Tanzanian patients. Interestingly, though not significant the opposite trend was observed in Ethiopian HIV patients, i.e., a trend of having higher plasma efavirenz concentration during rifampicin based anti-TB cotreatment particularly in CYP2B6*6 carriers.

CYP2B6, the major efavirenz metabolizing enzyme, is inducible by both efavirenz and rifampicin, and concomitant administration of the two drugs may result in additive effect to increase efavirenz clearance and reduce efavirenz plasma concentration. Indeed, studies in the white population reported that rifampicin co-treatment significantly reduced efavirenz plasma concentration (Lopez-Cortes et al., 2002). However, in both Ethiopians and Tanzanians TB-HIV patients, rifampicin co-treatment did not reduce efavirenz plasma concentration (**Figure 1**). Similar to our finding previous studies from other black and Asian populations reported no effect or paradoxically increased plasma efavirenz concentration during concomitant rifampicin based anti-TB therapy (Cohen et al., 2009: Gengiah et al., 2012: Mukonzo et al., 2013: Mukonzo et al., 2014a: Dhoro et al., 2015). This inconsistent finding of antiretroviral-antituberculosis drug interaction between white and black populations indicates the relevance of population differences in drug-interaction studies and the need for cautious extrapolation of finding from one population to another. Our result indicates that anti-TB co-medication reduced efavirenz clearance as shown by a trend of increased plasma efavirenz concentration overtime in both study populations. A similar finding in Ugandans and other population was reported (Gengiah et al., 2012: Mukonzo et al., 2013: Mukonzo et al., 2014a). Again, here *CYP2B6* genotype plays a significant role in antiretroviral-anti TB drug interactions. The increase in efavirenz plasma concentration was pronounced in those homozygous for the defective allele CYP2B6*6/*6 (**Figure 2**). Our finding is in line with previous reports where paradoxically elevated plasma efavirenz concentration by rifampicin co-treatment particularly in those with CYP2B6*6/*6 genotype than those subjects with the same genotype but not receiving anti-tuberculous therapy (Kwara et al., 2011).

In comparison to the TB-HIV patients, the HIV-only patients had higher mean efavirenz concentrations with statistically significant differences between the populations, where Tanzanian patients had much higher efavirenz concentrations compared to Ethiopian counterparts at both 4 and 16 weeks post-cART initiation (Ngaimisi et al., 2013). In the absence of rifampicin treatment, population differences plays a significant role in efavirenz pharmacokinetics, but its role diminishes in the presence of rifampicin co-treatment. Even among the rapid metabolizer groups with CYP2B6*1/*1 genotypes, plasma efavirenz concentration was higher in the TB-HIV patients compared to HIV only patients. This could be explained in part by drug interactions modified by other anti-tubercular agents such as isoniazid involving other metabolizing enzymes such *CYP2A6* and N-acetyl transferase type 2 (NAT2) as suggested previously (Bertrand et al., 2014: Court et al., 2014: Luetkemeyer et al., 2015). These data suggest that the inducing effect of rifampicin is counterbalanced by a concentration-dependent inhibitory effect of isoniazid on efavirenz clearance. Efavirenz metabolism *via* CYP2A6 is relevant particularly in those with slow CYP2B6 enzyme activity. CYP2B6 slow metabolizers and slow-acetylation NAT2 phenotype result in lowest efavirenz apparent clearance (Bertrand et al., 2014: Luetkemeyer et al., 2015). Isoniazid, a known inhibitor of CYP2A6, is metabolized by N-acetyltransferase-2 (NAT-2). Both CYP2A6 and NAT-2 are genetically polymorphic enzymes displaying wide between population variation in their enzyme activity (Djordjevic et al., 2013a: Djordjevic et al., 2013b). Apart from genetic factors, CYP2A6 and NAT-2 enzyme activities are also influenced by environmental factors (Podgorna et al., 2015: Aklillu et al., 2018). Hence genetic variation in CYP2A6 and NAT-2 may play a role in antiretroviral-anti-TB drug interactions by modulating plasma concentration of the inhibitor, isoniazid.

Regardless of population differences or anti-TB co-treatment, our result indicated no significant association of *CYP2B6* genotype or efavirenz plasma concentration with immunologic outcomes. Our finding is in line with previous studies that reported no association between plasma efavirenz concentration and virologic outcome (Dickinson et al., 2015: Mukonzo et al., 2016). Lack of association between efavirenz plasma concentration and CD_4_ recovery or virologic decay may indicate that plasma efavirenz concentration at 600 mg daily dose is way above the threshold for virologic failure (Mukonzo et al., 2014b: Mukonzo et al., 2016).

Multiple factors play a role in the pharmacokinetic variability of efavirenz including population variation. This study has shown differences in allele frequencies between Tanzanians and Ethiopians, and these differences may contribute to the pharmacokinetic variability when patients are treated with efavirenz and rifampicin among TB-HIV co-infected patients and more so in a similar population on efavirenz-based cART alone. This re-affirms that between population variations in drug exposures often occur, which leads to variations in clinical outcomes between different populations. Thus, knowing genetic variations in a genetically and culturally diverse African population is vital to optimizing treatment response.

The study has some limitations as we have not genotyped for other *CYP2B6* defective variant alleles such as *c.785A > G* and CYP2B6*18 (c.983T > C) that influence efavirenz pharmacokinetics (Dhoro et al., 2015). In this study, we genotyped for *c.516 G > T,* the most common *CYP2B6* defective variant SNP in the black African population. *c.516 G > T* is in strong linkage disequilibrium with other *CYP2B6* variant alleles, including c.*785A > G* (Mukonzo et al., 2009). Hence *c.516 G > T* can serve as CYP2B6*6 allele identifying SNP, a tag SNP for other relatively rare haplotypes, and to define clinically relevant inter-individual variability in CYP2B6 activity. Nevertheless, CYP2B6*18 (*c.983T > C*) is absent in Ethiopians (Habtewold et al., 2015), but it is present in Tanzanians with 9.3% allele frequency (Maganda et al., 2016: Mutagonda et al., 2017). Thus, a lack of genotyping for *CYP2B6 c.983T > C* among Tanzanians is our study limitation.

## Conclusion

We report the importance of pharmacogenetic and population variation for efavirenz pharmacokinetics, auto-induction, immunologic outcome, and interaction between antiretroviral anti-tuberculosis drugs. The extent of efavirenz auto-induction over time, plasma exposure, and immunologic outcome vary between populations, partly due to *CYP2B6* genotype and antituberculosis cotreatment. *CYP2B6* genotype is the primary determinant of plasma efavirenz concentrations regardless of population and anti-TB co-medication. Population differences plays a significant role in determining efavirenz plasma concentration and immunologic outcomes particularly in the absence of anti-TB cotreatment, but not during concomitant anti-TB treatment. Our findings underscore the importance of population variations and environment factors in addition to pharmacogenetic variation for efavirenz pharmacokinetics and dose optimization strategy in sub Sharan African population.

## Data Availability Statement

The raw genotyping data underlying the conclusions of this article are not publicly available as permission to do so was not included in the protocol approval granted by the ethics committee. The raw data supporting the conclusions of this article will be made available by the authors, without undue reservation, to any qualified researcher.

## Ethics Statement

The study received ethical approval from the Institutional Review Board (IRB) of the Muhimbili University of Health and Allied Sciences in Dar es Salaam, Tanzania, by the IRB of the Faculty of Medicine, Addis Ababa University and the Ethiopian National Ethics Review Committee and by the IRB of Karolinska Institutet in Stockholm, Sweden. The patients/participants provided their written informed consent to participate in this study.

## Author Contributions

EA, OM, EM, and JB contributed to the conception and design of the study. SM, WA, AH, EM, GY, EA, OM, and EM conducted the clinical study and data collection. EA, AH, EN, and JB conducted plasma drug quantification and genotyping. SM, EA, and CS conducted data analysis and interpretation. EA and SM wrote the draft manuscript. All authors contributed and approved the final article.

## Funding

This study was supported by grants from the European and Developing Countries Clinical Trial Partnership (grant number CT.2005.32030.001) Swedish Research Council (Vetenskapsrådet) grant number: 2015-03295. The research analyzed and reported in this publication was supported by the Fogarty International Center of the National Institutes of Health under Award Number D43 TW009775. The content is solely the responsibility of the authors and does not necessarily represent the official views of the National Institutes of Health.

## Conflict of Interest

The authors declare that the research was conducted in the absence of any commercial or financial relationships that could be construed as a potential conflict of interest.
